# Comparative Evaluation of Germination Methods on the Nutritional and Sensory Profile of *Coix*

**DOI:** 10.3390/foods15111925

**Published:** 2026-05-29

**Authors:** Qing Hu, Nan Li, Hongxiao Liu, Chao Tang, Suyang Duan, Fengzhong Wang, Lina Liu, Sha Yang, Xuyan Dong

**Affiliations:** 1 Key Laboratory of Novel Food Resources Processing, Ministry of Agriculture and Rural Affairs, Institute of Food & Nutrition Science and Technology, Shandong Academy of Agricultural Sciences, 23788 Gongye North Road, Jinan 250100, China; hqingu@163.com (Q.H.); linanyd@126.com (N.L.); liuhongxiao0621@163.com (H.L.); duansuyang16@163.com (S.D.); wangfengzhong@sina.com (F.W.); 2 College of Food Science and Engineering, Qingdao Agricultural University, Qingdao 266109, China; dongxuyan@qau.edu.cn; 3 Key Laboratory of Agro-Products Processing, Institute of Food Science and Technology, Chinese Academy of Agricultural Sciences, Ministry of Agriculture and Rural Affairs, Beijing 100193, China; 4 College of Food Science and Engineering, Yangzhou University, Yangzhou 225127, China; 008155@yzu.edu.cn; 5 National Center of Technology Innovation for Comprehensive Utilization of Saline-Alkali Land, Dongying 257092, China

**Keywords:** ultrasonic treatment, heat stress, germination, electronic tongue, functional foods

## Abstract

*Coix* has gained significant research interest for its medicinal and nutritional value, yet its characteristic bitterness limits food applications. To enhance its utilization in the food industry, this study examined the impact of conventional water immersion, ultrasound-assisted immersion (10, 15, and 20 min) and heat-treated immersion (40, 50, and 60 °C) on the nutritional profile and taste properties of *Coix*. Germinated *Coix* showed reduced starch, fat, and phytic acid content, but increased protein, γ-aminobutyric acid (from 38.05 to 58.00–116.90 mg/100 g, *p* < 0.05), dietary fiber, soluble sugars, total phenolics, and DPPH radical scavenging activity (*n* = 5). Germination introduced palmitoleic, linolenic, arachidonic, myristic, and arachidic acids to *Coix*, increasing total amino acids and umami taste activity value from 88.09 to 104.50–141.39 (*p* < 0.05). The reduced bitterness and astringency of germinated *Coix* may be associated with lower linoleic acid content and higher levels of palmitoleic acid, aspartic acid, and glutamic acid. Ultrasound-assisted immersion for 20 min was identified as the optimal condition for enhancing GABA and amino acid contents while reducing bitterness, thereby broadening the food applications of *Coix*.

## 1. Introduction

*Coix* (*Coix lacryma-jobi* L.), an herbaceous plant belonging to the Poaceae family [1], is widely cultivated in Asian countries such as China, India, and Myanmar because of its medicinal and edible value. Its seeds are widely used as both food and traditional medicinal materials. *Coix* is rich in protein, amino acids, oil [2], and dietary fiber, as well as various bioactive compounds such as sterols, lignans, phenolic compounds, alkaloids, etc. Previous studies have shown that *Coix* possesses multiple pharmacological activities, including anticancer [3], anti-inflammatory [4], glycemic regulation [5], hepatoprotective effects, and gut microbiota modulation [6]. Currently, *Coix* is primarily consumed as a key ingredient in multigrain porridges, with limited utilization in processed foods such as biscuits and beverages [7]. One major obstacle is the characteristic bitterness of shelled *Coix* seeds, which is thought to be associated with several classes of compounds, including polyphenols, fatty acids, amino acids, and possibly other low-molecular-weight metabolites. At present, the specific bitter contributors in *Coix* are still poorly characterized, and effective strategies for taste improvement remain limited. Evidence from other grains and legumes suggests that bitterness is often caused by multiple chemical groups rather than by a single compound. For example, bitterness in *Tartary buckwheat* has been mainly attributed to phenolic compounds such as quercetin, whereas bitterness in rye has been associated with phenolics, including pinoresinol and syringic acid, as well as small molecular weight peptides, amino acids, and fatty acids [8,9,10]. In faba beans, undesirable flavor attributes related to lipids, tannins, and other anti-nutritional components can be modified by germination, resulting in reduced bitterness and fewer off-odors [11]. These findings suggest that the bitterness of *Coix* may also arise from multiple secondary metabolites and that germination may be a promising approach for improving its taste. In addition to activating metabolic pathways that generate new bioactive precursors, germination alters grain structure and has been reported to improve the sensory properties of *sorghum* [12], *wheat-based soy sauce* [13], *rice noodles* [14] and other grain products. Collectively, these findings suggest that germination is a promising strategy for improving both the nutritional and sensory quality of grains.

Germination activates endogenous enzymes (e.g., amylase, protease, and lipase), enhancing the synthesis of nutritionally valuable components including soluble sugars, γ-aminobutyric acid (GABA) [15], and amino acids in *Coix*. Concurrently, this process reduces anti-nutritional factors such as phytic acid [16], while elevating phenolic compounds and other bioactive constituents, thereby augmenting antioxidant capacity [17]. These biochemical transformations not only improve nutritional profiles but may also modulate flavor attributes. Furthermore, germinated grains demonstrate potential health benefits: β-glucans and phenolics exhibit antitumor activity [18,19], while GABA, dietary fiber, plant sterols, and tocopherols contribute to hypoglycemic and hypolipidemic effects through lipid metabolism regulation [20,21].

Different germination methods may differentially influence seed physiology. The traditional water immersion method mainly activates metabolism through water absorption. Physical treatments, including microwave, ultrasound, pulsed electric field, and irradiation, have been reported to affect seed metabolism by altering membrane properties, enzyme activity, and related physiological responses [22]. Ultrasound-assisted immersion, for instance, weakens seed coats through acoustic cavitation, facilitating water uptake and dormancy breakage [23,24]. Such physical methods enhance germination rates while increasing reducing sugar content and antioxidant activity (e.g., DPPH/ABTS radical scavenging) [25]. The theoretical basis for stress-induced germination (e.g., heat stress, osmotic stress, or hypoxic stress) is that seeds activate cellular defense mechanisms to overcome dormancy under moderate stress, whereas excessive stress inhibits germination. Research has shown that *Physalis peruviana* seeds exhibited maximum germination at 20–30 °C under heat-treated immersion, with complete inhibition occurring at 40 °C, and osmotic potentials ≤ −0.9 MPa suppressed germination entirely [26]. Therefore, different germination methods have specific regulatory effects on the seed metabolic network through differentiated physicochemical pathways.

Current research on germinated *Coix* has mainly focused on nutritional enhancement achieved by conventional soaking, whereas the simultaneous improvement of sensory quality through alternative germination strategies remains insufficiently explored. To better exploit the edible and functional value of *Coix* and expand its use in the food industry, the present study compared the effects of water immersion germination, ultrasound-assisted immersion germination, and heat-treated immersion germination on the nutritional composition and taste characteristics of *Coix*. Particular attention was paid to changes in amino acid and fatty acid profiles and their potential associations with bitterness reduction. We hypothesized that ultrasound-assisted immersion and heat-treated immersion, as physical stress treatments, would differentially regulate seed metabolism compared with conventional water immersion, leading to distinct effects on GABA accumulation, antioxidant capacity, amino acid/fatty acid composition, and bitterness-related taste attributes. This work provides insight into the coordinated improvement of nutritional and sensory quality in *Coix* and offers a basis for its industrial utilization.

## 2. Materials and Methods

### 2.1. Materials

The *Coix* seeds (variety: *Coix* No. 2) were procured from the Guizhou Academy of Agricultural Sciences (Guiyang, China) in September 2024, sealed and stored at 4 °C. The starch content assay kit was from Solarbio (BC0700, Solarbio, Beijing, China). The enhanced BCA protein assay kit was from Beyotime (P0010S, Beyotime, Shanghai, China). A mixed standard of fatty acid methyl esters was purchased from Sigma-Aldrich (18919-1AMP, Sigma-Aldrich, St. Louis, MO, USA). The n-hexane standards (HPLC grade) were purchased from Kemio (1208, Tianjin, China). All chemicals were analytical-grade reagents sourced from China National Pharmaceutical Group Chemical Reagent (Shanghai, China).

### 2.2. Germination Process

Three germination treatments, water immersion (WI), ultrasound-assisted immersion (UI), and heat-treated immersion (HI), were applied to *Coix* seeds. The non-germinated sample (NG) was used throughout the study as the baseline control. Surface sterilization of all seeds was carried out with 0.1% NaOCl at a 1:5 (*w*/*v*) seed-to-solution ratio. For WI, sterilized seeds were soaked in 1:10 (*w*/*v*) distilled water for 12 h, placed on a culture dish with sterile gauze, germinated in a 30 °C climate-controlled incubator (DHP-9032, Yi Heng, Shanghai, China), and misted with distilled water every 12 h to maintain humidity for germination. After 72 h of germination in the dark, the germinated *Coix* was washed with distilled water, dried to a constant weight in a 40 °C oven (GZX-9070MBE, Bo Xun, Shanghai, China), and designated as WI samples. For UI, ultrasound-assisted immersion was carried out using a constant-temperature ultrasonic device (SB25-12DT, Xin Zhi, Ningbo, China) at 25 ± 1 °C, 40 kHz, and 600 W for 10, 15, or 20 min before soaking. Water temperature was monitored with thermometers, and ice packs were used when necessary to maintain the target temperature. For HI, sterilized seeds were subjected to 15 min of hydrothermal treatment in a water bath at 40 °C, 50 °C, or 60 °C (with a temperature tolerance of ±1 °C), labeled HI-40, HI-50, and HI-60, before proceeding to soaking and germination. All dried *Coix* was ground into fine flour using a mechanical grinder (A11, IKA, Staufen, Germany), samples were sieved (100-mesh) and kept at −20 °C until subsequent analysis. All experiments were performed in quintuplicate (*n* = 5). The UI durations (10, 15, and 20 min) and HI temperatures (40, 50, and 60 °C) were selected on the basis of preliminary screening trials in *Coix*. These conditions represented low-, medium-, and relatively strong treatment levels that were sufficient to induce measurable physiological responses while still permitting germination, and were therefore considered suitable for comparative evaluation rather than as fully optimized industrial parameters [27,28,29].

### 2.3. Determination of Germination Rate

*Coix* seed germination was monitored at 12 h intervals using batches of 30 seeds. At least five batches were set up, and the germination percentage was calculated using Equation (1):

(1) $$\mathrm{Germination}\;\mathrm{rate}\;(\%)\;=\;\frac{G_{s}}{T_{s}}\;\times \;100$$

where G_s_ is the number of germinated seeds and T_s_ is the total number of seeds.

### 2.4. Determination of Starch, Protein, and Fat Contents

Following the operating instructions, the starch (BC0700, Solarbio, Beijing, China) and protein (P0010S, Beyotime, Shanghai, China) assay kits were used to determine the starch and protein content in *Coix*, respectively. The fat content was extracted using petroleum ether in accordance with GB 5009.6-2016; National food safety standard-Determination of fat in food. China Standard Press, Beijing, 2016. All experiments were performed in quintuplicate (*n* = 5).

### 2.5. Determination of Total Soluble Sugar Content

Total sugar content was quantified using the anthrone–sulfuric acid method following Sebnem et al. [30], modified as follows: Glucose standards (0–80 μg/mL) were reacted with 5 mL anthrone reagent, incubated in a boiling water bath (LC-WB4, Lichen, Shanghai, China) for 10 min, and absorbance measured at 620 nm (Synergy H1, BioTek, Winooski, VT, USA) against a water blank. Total soluble sugar content was quantified using the calibration equation (Y = 0.0052X + 0.131, R^2^ = 0.9969). All experiments were performed in quintuplicate (*n* = 5).

### 2.6. Determination of Total Polyphenol Content

A modified method based on Khatun and Mollah [31] was employed to determine the total polyphenol content (TPC) in *Coix*. Briefly, *Coix* powder (1 g) was extracted with 10 mL ethanol (70%) and then subjected to 3 h of continuous shaking at 180 r/min (SHZ-A, Gao Ke, Henan, China), centrifuged (Xima Centrifuge Co., Ltd., Yangzhou, China) at 1800× *g* for 10 min to collect the clear liquid on top of sediment. For analysis, 100 μL of the extract was mixed with an equal volume of Folin-Denis reagent. After the reaction, 1 mL of 0.7 mol/L sodium carbonate solution was added. The absorbance was then measured at 750 nm. Ungerminated seed extracts were used as controls. Total phenolic content was determined employing a tannic acid standard curve (0–250 μg/mL; Y = 0.1114X + 0.0774; R^2^ = 0.9906), where X and Y represented concentration and absorbance, respectively. Assays were repeated five times, and results were expressed as mg tannic acid equivalent per gram of seed extract (*n* = 5).

### 2.7. Determination of DPPH Radical Scavenging Rate

A modified method from Nakagawa et al. [32] was used to assess DPPH radical scavenging activity. The assay was performed by mixing 0.5 mL of the test solution (concentrations: 25, 50, 100, 200, 400 mg/mL) with 4.5 mL of a 60 μmol/L DPPH solution, followed by a 30 min incubation under dark conditions at room temperature and subsequent measurement of the absorbance at 517 nm. The blank group was treated with methanol solution. All experiments were performed in quintuplicate (*n* = 5).

The DPPH radical scavenging rate was calculated using Equation (2):

(2) $$\mathrm{DPPH}\;\mathrm{radical}\;\mathrm{scavenging}\;\mathrm{activity}\;(\%)=\frac{D_{1}-D_{0}}{D_{1}}\;\times \;100$$

D_1_ and D_0_ were defined as the absorbance values of *Coix* sample and the control, respectively.

### 2.8. Determination of GABA

GABA content was determined according to the method of Sharma et al. [33] with some modifications. Following a 2 h extraction of homogenized samples (0.5 g *Coix* powder in 5 mL distilled water) that were centrifuged (8000× *g*, 5 min), 0.5 mL of the resulting supernatant was combined with 0.5 mL of 0.2 mol/L borate buffer (pH 9.0), 1 mL of 5% (*w*/*v*) phenol, and 0.5 mL of NaOCl (7% active chlorine). After vortexing and boiling (10 min), samples were shaken for 20 min, during which a blue-green chromophore was formed. Absorbance at 640 nm was measured after adding 1 mL of 60% ethanol. GABA content was quantified using the calibration equation (0–15 mg/mL, Y = 0.0099X + 0.0785, R^2^ = 0.9918) obtained from the standard curve of GABA. All experiments were performed in quintuplicate (*n* = 5).

### 2.9. Determination of Dietary Fiber

Dietary fiber content was determined using a modified version of the method described by Kanwar et al. [34]. Defatted coix powder (0.2 g; n-hexane-treated 1:5 *w*/*v*, 12 h) was suspended in acetic acid buffer (0.1 mol/L, pH 6, 4 mL) with α-amylase (20 μL), incubated at 96 °C for 1 h with stirring. After pH adjustment to 7.5 (NaOH), protease (20 μL) was added (60 °C, 30 min). Following pH shift to 4.5, amyloglucosidase (100 μL, ≥260 U/mL) was introduced (60 °C, 4 h). Precipitated fibers (80% ethanol addition) were collected by centrifugation (2000× *g*, 20 min), washed (80% ethanol/acetone), dried (40 °C), sieved (100-mesh), and weighed. The measurement was repeated five times (n = 5). Dietary fiber content was calculated by the following Formula (3):

(3) $$\mathrm{Dietary}\;\mathrm{fiber}\;\mathrm{content}\;(\%)\;=\;\frac{W_{1}}{W_{0}}\;\times \;100$$

where W_1_ is the total weight of the extracted residue and W_0_ is the initial weight of *Coix* before extraction.

### 2.10. Determination of Phytic Acid

The phytic acid content of *Coix* was determined as described by Nana et al. [35] with slight modifications. Briefly, phytic acid was extracted from 0.5 g of germinated *Coix* powder with 20 mL of 0.2 mol/L HCl on a 30 °C shaker (100 r/min, 3 h), followed by centrifugation (1800× *g*, 10 min). A 1 mL aliquot of the supernatant was transferred to a new tube (15 mL), mixed with 2 mL of 0.2 g/L ammonium ferric sulfate solution, and sequentially incubated in a 95 °C water bath (30 min) and an ice-water bath (20 min) before re-centrifugation. Subsequently, 1 mL of the resulting supernatant was combined with 1.5 mL of 1% bipyridine solution in a 5 mL tube, and the absorbance was measured at 519 nm against a distilled water blank. Phytic acid content was quantified using the standard curve (0–100 μg/mL, Y = 0.3643X + 0.0954, R^2^ = 0.998), with results as mg phytic acid per gram extract. All experiments were performed in quintuplicate (*n* = 5).

### 2.11. Determination of Free Amino Acids

Free amino acid levels were determined using an automatic amino acid analyzer (LA8080, Hitachi, Tokyo, Japan). Under a nitrogen atmosphere, a 0.1 g sample was treated with 10 mL of 0.02 mol/L hydrochloric acid and sealed. A 30 min shaking extraction of the combined solution was carried out at room temperature. Then, 1 mL of the mixture was taken, dried under nitrogen flow, and reconstituted with 1 mL of water. The redried residue was taken up in 0.02 mol/L hydrochloric acid to a final volume of 1 mL. Finally, the filtrate obtained from 0.22 μm membrane filtration was used for free amino acid analysis. The chromatographic column was a sulfonic acid cationic resin. Detection was carried out at 570 and 440 nm. The amino acid standard used was the H-type mixed amino acid standard solution from FUJIFILM Wako Pure Chemical Corporation (Osaka, Japan). The experiment was repeated five times (*n* = 5). The amino acid taste activity value (TAV) was calculated according to Formula (4):

(4) $$\mathrm{TAV}\;=\;\frac{C}{T}$$

where C is the concentration of the compound in the sample and T is its taste threshold.

### 2.12. Determination of Fatty Acids

A 0.1 g fat sample from germinated *Coix* was placed into a 10 mL centrifuge tube, and 3 mL of a 2% NaOH-CH_3_OH solution was added. The sealed tube was subjected to a 25 min incubation in an 80 °C water bath. After the sealed tube was cooled, 1.5 mL of a 14% BF_3_-CH_3_OH solution was added to the tube, which was then resealed and heated for an additional 7 min. After cooling, 2 mL of n-heptane was added and the mixture was vortexed thoroughly. Subsequently, 1 mL of saturated NaCl solution was added. After being thoroughly mixed and left to stand, the formation of different layers was observed. The upper organic layer was transferred to a clean microcentrifuge tube, and a small amount of anhydrous Na_2_SO_4_ was added. After the mixture had been vigorously shaken for 1 min, it was allowed to stand for 5 min to ensure that water was completely removed from the organic phase. One milliliter of the supernatant was filtered through a 0.22 μm organic membrane filter.

Analysis was performed on an Agilent GC (7890B, Agilent, Santa Clara, CA, USA) system equipped with a DB-23 capillary column (60 m × 0.25 mm, 0.25 µm). The injector temperature was set at 250 °C with a split ratio of 50:1. An injection volume of 1 μL was used, and the carrier gas flow rate was maintained at 20 mL/min. The oven temperature program was as follows: the temperature was held at 50 °C for 1 min, was ramped to 175 °C at 25 °C/min and held for 1 min, then was increased to 230 °C at 4 °C/min and held for 5 min. All assays were repeated five times (*n* = 5).

### 2.13. Determination of Taste Properties

Samples were assessed using an electronic tongue (SA402B, Insent, Atsugi, Japan) based on a modified method from Zhao et al. [36]. A total of 5 g of the sample was weighed, diluted 20 times with distilled water, and homogenized. The mixture was then centrifuged at 8000× *g* for 5 min. The supernatant was placed in a sealed glass device for measurement. Before the sample was tested, the test sensor and the reference sensor were activated for 24 h. After they were activated, about 35 mL of solution was added to the corresponding test cup, and the sample collection time was set to 120 s. Data were collected every 1 s, and for each sample, the output value was calculated as the average of the data recorded during the final 30 s of measurement. The electronic tongue detection experiment was equipped with five sensors: umami sensor (AEE), saltiness sensor (CT0), sour sensor (CA0), bitter sensor (C00), and astringent sensor (AE1). The electronic tongue was used to evaluate umami, saltiness, sourness, bitterness, and astringency in the samples. The electronic tongue system was calibrated with a standard solution, and processes such as self-inspection, initialization, and correction were completed before each sample was measured. The data were processed using the system’s built-in software (Ver1.0.0.5), and each sample was measured five times (*n* = 5).

### 2.14. Statistical Analysis

Data from five replicates are presented as mean ± standard deviation (SD). Normality and homogeneity of variance were assessed using the Shapiro–Wilk and Levene tests, respectively. Differences among treatments were analyzed by one-way analysis of variance (ANOVA), followed by Duncan’s multiple range test for post hoc pairwise comparisons when the ANOVA was significant (*p* < 0.05). Spearman’s rank correlation analysis was used to assess associations between chemical composition and electronic tongue responses. Statistical analyses were performed using SPSS Free Edition 21.0 (SPSS Corporation, Chicago, IL, USA), and figures were generated using Origin 2021 (OriginLab, Northampton, MA, USA).

## 3. Results and Discussion

### 3.1. Germination Rate

**Figure 1:**
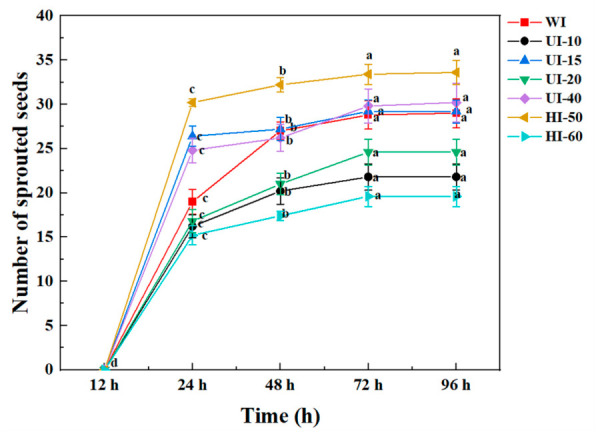
*Coix* seeds germination at 12, 24, 48, and 72 h. Different letters in the same subgraph indicated significant differences (p < 0.05).

**Figure 2:**
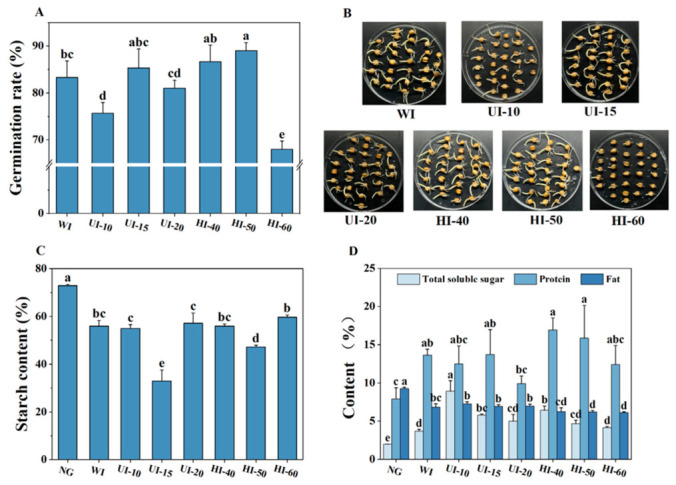
Germination rate (**A**), seed germination status (**B**), starch content (**C**), and total soluble sugar, protein and fat content (**D**) of *Coix* germinated at 72 h (*n* = 5). NG: non-germinated *Coix*; WI: water immersion germinated *Coix*; UI-10, UI-15, UI-20: *Coix* germinated by ultrasound-assisted immersion for 10, 15, and 20 min, respectively; HI-40, HI-50, HI-60: *Coix* germinated by soaking *Coix* in 40, 50, and 60 °C water for 15 min, respectively. Different letters in the same subgraph indicated significant differences (*p* < 0.05).

Germination rate is commonly used as an indicator of seed quality and performance [37]. Using UI-10 as an example (Figure 1), almost no germination of *Coix* seeds was observed at 12 h. The number of germinated seeds at 24 h was 16.20 ± 1.30, increasing to 20.20 ± 1.48 at 48 h, and further rising to 21.80 ± 1.48 at 72 h. At 96 h, the value remained at 21.80 ± 1.48. These results indicate that the number of sprouts between 72 h and 96 h was nearly identical, with no significant difference between the two time points (*p* > 0.05). Consequently, germination increased markedly after 24 h and showed no further increase between 72 and 96 h under this example condition. Therefore, the germination data at 72 h were used for subsequent statistical analysis. As shown in Figure 2A, heat-treated immersion at 50 °C (HI-50: 89.0 ± 1.73%) and ultrasound-assisted immersion for 15 min (UI-15: 85.33 ± 4.04%) resulted in numerically higher germination rates compared to the WI group (83.3 ± 3.51%). Furthermore, the germination rate of HI-50 was numerically higher than that of UI-15. In contrast, prolonging the ultrasound-assisted immersion time or increasing the heat-treated immersion temperature led to a decrease in germination rates. Figure 2B showed that, under HI-60, sprout growth was visibly slower and bud length was shorter than in the other germinated treatments. Gong et al. [38] also found that the germination rate and embryonic root length of *corn* seeds increased by 10.40% and 230.50%, respectively, after ultrasound-assisted immersion. Goff et al. [39] also found that appropriate temperatures (20–70 °C) promoted *pecan* germination, whereas excessively high temperature (80 °C) inhibited it. Germination performance depended on the balance between stimulation and damage. Moderate ultrasound-assisted immersion may enhance seed hydration and gas exchange by creating microstructural defects on the seed surface, thereby accelerating metabolic activation and dormancy release [40]. However, prolonged ultrasound exposure may impose excessive cavitation-related mechanical stress on the embryo and surrounding tissues, resulting in reduced viability [41]. A similar trade-off was observed under HI conditions: moderate heat treatment may accelerate metabolic activation during early imbibition, whereas excessive temperature may induce thermal inhibition or secondary dormancy and impair subsequent sprout development [42]. Al-Gharaibeh et al. [43] found that 4 °C broke the dormancy of *Rosa pulverulenta* seeds and promoted germination, whereas temperatures beyond this range inhibited germination. They also reported that higher temperatures (20–25 °C) induced secondary dormancy and reduced germination performance. This interpretation is supported by the comparatively high germination rate under HI-50 and the reduced germination performance and bud length under HI-60, indicating that the effect of physical pretreatment on *Coix* germination is intensity-dependent rather than uniformly beneficial.

### 3.2. Changes in Starch, Protein, and Fat Content

Seed germination was initiated and maintained by the energy generated from the degradation of stored substances [44]. The starch, protein, and fat contents of *Coix* under different germination treatments are shown in Figure 2C,D. Compared with NG, several germination treatments showed lower starch and fat contents and a higher protein content, although the magnitude of change depended on the treatment. Among the treatments, UI-15 (32.98 ± 4.61%) and HI-50 (47.22 ± 0.77%) showed lower starch contents than NG (72.9 ± 0.56%). In addition, the HI treatments showed higher protein contents than the UI treatments and NG, with the highest value reaching 16.93 ± 1.58%. Compared with NG samples, protein increased from 7.90% to 9.90–16.93%, and fat decreased from 9.26% to 6.10–7.26%. These results were consistent with previous studies demonstrating that ultrasound-assisted immersion decreased starch content in *brown rice* and germinated rice [45]. This pattern of increased protein content and decreased fat content is consistent with the findings of Yu et al. [46], who reported that ultrasound-assisted germination improved the nutritional quality of red *kidney beans*. Ultrasound may enhance water absorption by creating microstructural defects on the seed coat and outer layers. Meanwhile, the compositional changes in starch, protein, and fat also provide a metabolic basis for subsequent changes in functional and taste-related compounds. During germination, starch and lipids are mobilized to provide both energy and carbon skeletons for embryo growth, while protein turnover contributes to the release of free amino acids [47]. The breakdown of these storage compounds also provides precursors for the accumulation of soluble sugars, free amino acids, and GABA, which are often elevated in germinated seeds. The increase in total protein content in germinated seeds may be attributable to the enzymatic conversion of insoluble proteins [48]. Therefore, the lower starch and fat contents and the higher protein content observed in several germinated treatments should not be viewed as isolated compositional changes, but rather as indicators of intensified reserve mobilization.

### 3.3. Changes in Total Soluble Sugar Content

Soluble sugars are important osmotic regulators in plants and might play a crucial role in maintaining protein stability. Soluble sugars have been reported to play a key regulatory role in seed germination and early plant establishment [49,50,51]. Sprouting has been reported to increase the total soluble sugar content of *Coix* [52]. As shown in Figure 2D, the total soluble sugar contents were as follows: NG had 1.98 ± 0.02%; WI had 3.68 ± 0.23%; HI ranged from 4.14 ± 0.13% to 6.42 ± 0.55%; UI ranged from 4.97 ± 0.90% to 8.92 ± 1.35%. Total soluble sugar content increased after germination, with UI and HI showing numerically higher values than WI. The extent of increase, however, depended on the treatment condition. In particular, the highest soluble sugar content was observed in UI-10, which differed significantly from WI (*p* < 0.05). The increase in total soluble sugar content may be attributed to enhanced enzymatic activity during germination, during which starch is hydrolyzed by amylase into soluble sugars [53,54].

### 3.4. Changes in Total Polyphenol Content

**Figure 3:**
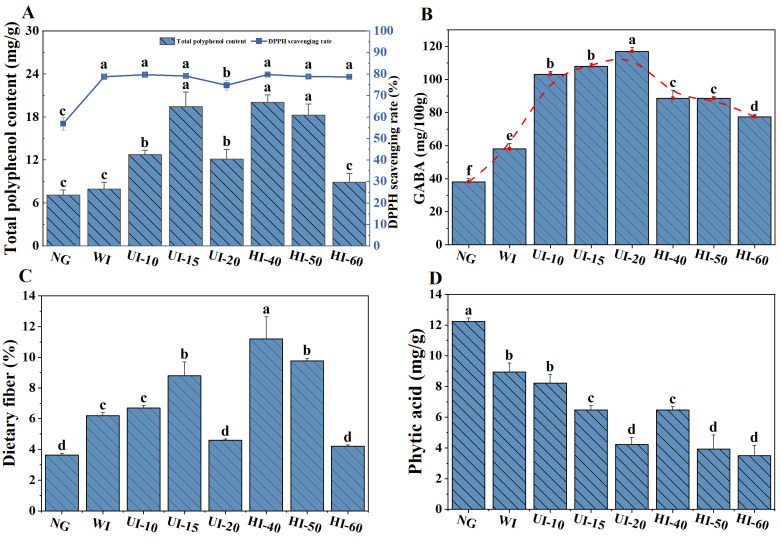
Total phenol and DPPH clearance rate (**A**), GABA content (**B**), dietary fiber content (**C**), and phytic acid content (**D**) of *Coix* germinated at 72 h (*n* = 5). NG: non-germinated *Coix*; WI: water immersion germinated *Coix*; UI-10, UI-15, UI-20: *Coix* germinated by ultrasound-assisted immersion for 10, 15, and 20 min, respectively; HI-40, HI-50, HI-60: *Coix* germinated by soaking *Coix* in 40, 50, and 60 °C water for 15 min, respectively. Different letters in the same subgraph indicated significant differences (*p* < 0.05).

Phenolic compounds are widely recognized for their antioxidant properties in plants [55]. Polyphenols were involved in various functions of plants, including defense against pathogen invasion and UV protection [56]. According to Figure 3A, the total polyphenol content of *Coix* increased from 7.10 mg/g to 7.95–20.06 mg/g under different germination conditions. A similar increase in total phenolic content during germination has also been reported in rice and oats [57]. This phenomenon may be related to enzyme activation and grain structural modification during germination, which can promote the accumulation of endogenous phenolic compounds and enhance antioxidant activity [58]. The total phenolic content of ultrasound-assisted immersion germination (UI) and heat-treated immersion germination (HI) was higher than that of water immersion germination (WI). Among them, the total polyphenol content of UI-15 and HI-40 samples was more than twice that of WI samples. However, prolonged ultrasound-assisted immersion and higher heat-treatment temperatures led to a decrease in total phenolic content. The negative effect of excessive heating temperature (180 °C) on total phenolic content and antioxidant capacity was confirmed in the study by Ross et al. [59] on grape seed flour.

### 3.5. Changes in DPPH Radical Scavenging Rate

The DPPH radical scavenging rate is commonly used as an indicator of antioxidant activity [60]. As shown in Figure 3A, germination enhanced the DPPH radical scavenging capacity of *Coix*. Xu et al. [61] also reported that the DPPH radical scavenging rate of *Coix* increased after germination using the traditional water immersion method. Some germinated treatments differed significantly from WI (*p* < 0.05), whereas no significant differences were observed among most of the remaining germinated samples, except for UI-20. The radical scavenging rate of NG samples was 56.90 ± 2.94%. The radical scavenging rates of UI-10 (79.69 ± 0.65%) and HI-40 (79.74 ± 0.92%) were higher than that of NG. Similar increases in total phenolic content and DPPH scavenging activity after germination have also been reported in *Cajanus* [62], *red beans* [63], and *corn* [64].

### 3.6. Changes in GABA Content

**Figure 4:**
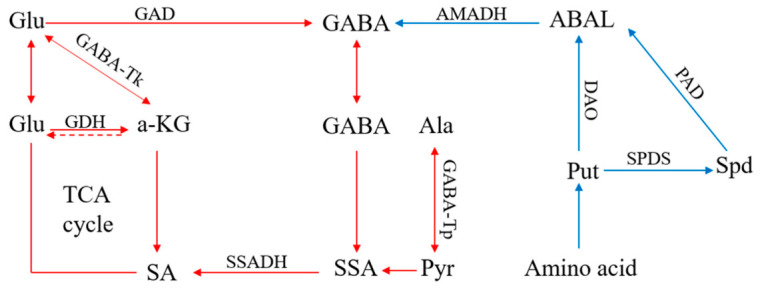
GABA synthesis pathway. The red pathway indicates the GABA shunt; the blue pathway represents the polyamine degradation pathway; the red dashed arrow represents that the two can transform into each other under certain conditions. In the figure, Glu represents glutamic acid; Ala, alanine; Pyr, pyruvic acid; GAD, glutamate decarboxylase; GABA-Tp, pyruvate-dependent γ-aminobutyric acid transaminase; GABA-Tk, α-ketoglutarate-dependent γ-aminobutyric acid transaminase; SSA, succinic semialdehyde; SSADH, succinate semialdehyde dehydrogenase; SA, succinic acid; GDH, glutamate dehydrogenase; a-KG, a-ketoglutaric acid; Put, putrescine; DAO, diamine oxidase; ABAL, 4-aminobutyraldehyde; SPDS, spermidine synthase; Spd, spermidine; PAO, polyamine oxidase; AMADH, aminoaldehyde dehydrogenase.

Gamma-aminobutyric acid (GABA) is a bioactive amino acid that has been associated with antihypertensive and other health-promoting effects [33]. As shown in Figure 3B, germination markedly altered the GABA content of *Coix*. Compared with NG, all germinated samples showed higher GABA contents. This observation is consistent with previous reports in other germinated grains and legumes [65]. Because glutamate is the direct precursor of GABA, the increase in GABA is closely related to the activation of endogenous amino acid metabolism during germination [66]. The GABA content was 38.05 mg/100 g in NG, 103.00–116.90 mg/100 g in UI samples, and 77.40–88.55 mg/100 g in HI samples. GABA content increased with prolonged ultrasound-assisted immersion time. A possible explanation, supported by previous studies on ultrasound-assisted germination, is that acoustic cavitation may increase seed coat permeability and facilitate water uptake during early germination, thereby promoting metabolic activation. As illustrated in Figure 4, under these conditions, glutamate, the direct precursor of GABA, accumulated to higher levels in UI-treated samples, particularly in UI-20, in parallel with the highest GABA content. This pattern suggests that the elevated GABA content in UI-treated *Coix* may be associated with greater substrate availability and enhanced GABA-related metabolism. In addition, the concurrent increase in Ala suggests broader rearrangement of amino acid metabolism, consistent with intensified metabolic turnover under ultrasound-assisted germination. In contrast, GABA content in HI-treated samples declined as pretreatment temperature increased, although it remained above the WI level. This indicates that moderate thermal pretreatment may still stimulate germination-associated metabolism, whereas excessive heat may compromise GABA accumulation. One plausible mechanism is that higher temperatures may reduce the favorable enzymatic conditions for endogenous GAD-mediated conversion of glutamate to GABA and promote leakage of water-soluble precursors during imbibition. Therefore, the different GABA responses between UI and HI may reflect different modes of physical regulation, with ultrasound favoring metabolic activation and precursor conversion, whereas excessive heat appears to exert a stronger inhibitory effect on the processes underlying GABA accumulation [47].

### 3.7. Changes in Dietary Fiber Content

Adequate dietary fiber can reduce dietary energy density, slow food intake, promote satiety, and help prevent excessive fat accumulation [67]. Dietary fiber has been associated with improved bowel function and reduced serum cholesterol levels [68,69]. Dietary fiber content showed a treatment-dependent response to germination (Figure 3C). Under some conditions, germination increased dietary fiber content, whereas higher heat-treatment temperatures reduced it [70,71]. Ultrasound-assisted immersion germination could increase dietary fiber content [72]. The dietary fiber content of *Coix* initially increased and then decreased as ultrasound-assisted immersion time was prolonged. As the temperature of HI increased, the dietary fiber content gradually decreased and eventually fell below the water immersion germination content (*p* < 0.05). This may be attributed to heat-induced starch gelatinization, which may affect the retention or dissolution of dietary fiber [73]. However, high temperatures may cleave glycosidic bonds in dietary fiber, leading to fiber degradation [74]. These results suggest that appropriate ultrasound-assisted immersion and heat-treated immersion conditions can increase the dietary fiber content of *Coix*.

### 3.8. Changes in Phytic Acid Content

A high phytic acid content may adversely affect mineral absorption and utilization [75]. As shown in Figure 3D, the phytic acid content decreased in WI, UI, and HI samples compared with NG. A decrease from 12.23 mg/g to 3.50–8.94 mg/g was observed. Among the germinated treatments, both UI and HI showed lower phytic acid contents than NG. Ultrasound-assisted immersion time and heat-treatment temperature also affected phytic acid content. The lowest numerical values were observed in UI-20 and HI-60. Although HI-60 treatment minimizes phytic acid content, it concurrently decreases seed vigor and germination capacity. Hence, when the final product aims to retain high biological germination activity (e.g., for use in sprouted grains, seed propagation, or the production of viable sprouts), the application of HI-60 treatment is not advisable. Previous studies have shown that phytic acid content decreases with increasing temperature. Fukushima et al. [76] found that the reduction in phytic acid in *brown rice* was related to the increase in soaking temperature. Swasti [72] similarly reported that the decrease in phytic acid content in germinated brown rice was associated with ultrasound-assisted immersion. From a mechanistic perspective, ultrasound treatment may facilitate phytic acid reduction by improving water penetration and increasing the accessibility of endogenous phytase to its substrate under moderate conditions. These combined effects may help explain why UI samples, especially UI-20, showed a greater reduction in phytic acid content. Germination promoted the activity of phytase, which suggested that the decrease in phytic acid levels might be a consequence of the enhanced activity [77]. In contrast, heat-treated immersion may reduce phytic acid through phytase-related degradation and/or thermal destabilization of phytic acid-containing complexes. These interpretations remain inferential because phytase activity and cellular permeability were not directly measured in the present study.

### 3.9. Changes in Free Amino Acids

#### 3.9.1. Amino Acids Content

The nutritional quality and taste of food are strongly influenced by amino acid composition and content [78,79]. After germination, the essential amino acid and total amino acid content of *Coix* increased by 32.11% and 37.43%, respectively (as shown in Table 1). Ukpong et al. [80] also reported an increase in essential amino acid content in germinated brown rice. Germination activated proteases, which led to protein breakdown and the generation of new enzymes. These enzymes may facilitate amino acid release, improving amino acid content [81]. UI and HI samples exhibited higher amino acid contents, possibly because external stress during germination activated endogenous enzymes and promoted amino acid accumulation in *Coix* seeds. Compared with WI samples, UI and HI samples showed greater contents of both essential amino acids (EAAs) and total amino acids (TAAs). Among these treatments, *Coix* seeds subjected to 20 min of ultrasound-assisted immersion (UI-20) displayed the highest essential amino acid content. Germination increased the contents of glutamic acid (Glu) and alanine (Ala), which may be related to the higher GABA content observed in Figure 3B. Glu is the direct precursor of GABA, whereas Asp may be associated with broader amino acid rebalancing during germination. Their concurrent increase suggests a general enhancement of nitrogen metabolism in germinated *Coix*. Meanwhile, numerous phenolic metabolites are known to be biosynthetically related to phenylalanine (Phe) and tyrosine (Tyr). The decreased Phe and Tyr contents observed in germinated *Coix* may be associated with the increased accumulation of phenolic compounds shown in Figure 3A [82].

#### 3.9.2. Taste Activity Value (TAV) of Amino Acids

Taste activity value (TAV) refers to the ratio of the concentration of a taste-active compound in a sample to its taste threshold. Different amino acids possess distinct taste characteristics and thresholds. Taste threshold values were obtained from the books Taste Chemistry and Flavor Chemistry [83,84]. When TAV > 1, the compound is considered to contribute to taste perception; when TAV < 1, its contribution is considered limited. As shown in Table 2, all detected amino acids had TAVs greater than 1, indicating that they contributed to the taste profile of *Coix*. Glu showed the highest TAVs (81.13–131.39), followed by Ala (17.24–25.97), Val (13.69–18.63), His (12.40–15.51), Arg (12.21–15.81) and Phe (6.86–8.93). Compared with NG, germinated samples generally showed higher TAVs for umami amino acids (104.50–141.41), whereas the changes in bitter and sweet amino acids were more moderate. Accordingly, umami remained the dominant amino-acid-derived taste attribute in *Coix*. Chen [2] reported that bitterness in brown rice noodles could be reduced through germination and explained that it might be neutralized by soluble sugars and sweet amino acids, both of which came from the breakdown of starch and protein during germination. Therefore, germination enhanced the amino-acid-derived taste activity of *Coix*, with Glu remaining the dominant flavor amino acid and umami representing the major sensory advantage.

### 3.10. Changes in Fatty Acid Composition

Various unsaturated fatty acids in grains have been associated with reduced blood lipid levels and improved insulin sensitivity [85]. As shown in Table 3, four fatty acids were detected in NG, whereas nine fatty acids were detected after germination. The appearance of five additional fatty acids may be attributed to lipase-mediated release of esterified fatty acids from triglycerides during germination. With the activation of lipase, triglycerides were hydrolyzed into free fatty acids, which changed the composition of fatty acids and provided energy and carbon skeleton for seedling growth [86]. The lipid profile of non-germinated *Coix* was dominated by Palmitic, Stearic, Oleic, and Linoleic acids. After germination, five types of fatty acids (3 unsaturated and 2 saturated fatty acids) were newly produced, namely Myristic acid, Palmitoleic acid, Linolenic acid, Arachidic acid, and Arachidonic acid. Among these, linolenic acid and arachidonic acid are nutritionally important polyunsaturated fatty acids. Oleic acid accounted for the highest proportion among the unsaturated fatty acids, reaching approximately 47% after germination, followed by Linoleic acid. Palmitic acid accounted for the highest proportion among the saturated fatty acids. After germination treatment, WI, UI-10, and HI-50 treatments not only increased the relative content of unsaturated fatty acid Oleic acid, but also promoted the increase in Linolenic acid content. Jimenez et al. [87] studied the fatty acids in Quinoa after germination and found that the content of Oleic acid and Linoleic acid increased. The relative content of saturated fatty acids such as Stearic acid decreased, possibly because fatty acid degradation during germination provided energy and metabolic intermediates for seedling growth through β-oxidation and related pathways [88]. In addition, Stearic acid can be converted to Oleic acid by stearoyl-ACP desaturase [89], suggesting that a decrease in stearic acid may be accompanied by an increase in Oleic acid. As shown in Table 3, oleic acid increased and stearic acid decreased after germination, which is consistent with the proposed conversion relationship between these two fatty acids. By contrast, the relative content of palmitic acid decreased only in the UI-15 treatment. Among the three germination conditions, the UI-15 *Coix* group was characterized by an increase in unsaturated fatty acids and a concurrent decline in saturated fatty acids. Therefore, germination conditions clearly influenced the fatty acid profile of *Coix* [90].

### 3.11. Changes in Taste Properties

As shown in Table 4, sourness, astringency, and saltiness did not differ significantly among treatments (*p* > 0.05). The lowest bitterness response was observed in UI-20, which was significantly lower than that of NG, whereas the other germinated samples showed intermediate values. Umami responses differed only modestly among treatments; UI-15 showed the lowest value, while HI-60 showed the highest value. Richness was higher in UI-15 than in NG and WI, whereas the remaining treatments showed intermediate values. Taken together, the electronic tongue results suggest that germination did not uniformly alter all taste attributes, but ultrasound-assisted immersion treatment for 20 min was associated with the lowest bitterness response under the tested conditions. This finding is consistent with reports of bitterness reduction in germinated quinoa bread [91], as well as in buckwheat- and brown-rice-based products [92]. The observed taste improvement may be explained not only by the possible degradation of bitterness-related compounds during germination, but also by changes in the composition of taste-active metabolites [93]. In particular, the increases in Asp (6.96 to 9.10–10.02 mg/g) and Glu (24.34 to 28.62–39.41 mg/g) indicate that the sensory improvement of germinated *Coix* may partly result from a rebalancing of taste perception, in which stronger umami-related inputs reduce the relative prominence of bitterness. Taste activity value (TAV) analysis identified Glu and Asp as major flavor contributors (Table 2), while the electronic tongue results suggested increased taste richness after germination (Table 4). It should be noted that the bitterness-reduction effect observed here may be influenced by subsequent thermal processing in practical applications. Thermal processing can lead to denaturation and inactivation of enzyme proteins, thereby significantly reducing or even eliminating the ability of enzymes to degrade bitter compounds. Therefore, if the product undergoes thermal processing, the contribution of enzymatic degradation to bitterness reduction may be weakened. Whether the masking effect of umami amino acids can be maintained will depend on the processing intensity and the food matrix. Future studies should evaluate the extent of bitterness recovery under different thermal processing conditions (e.g., temperature and time) to assess the stability of this debittering effect in food processing.

### 3.12. Correlation Analysis Between Amino Acids and Taste Properties

**Figure 5:**
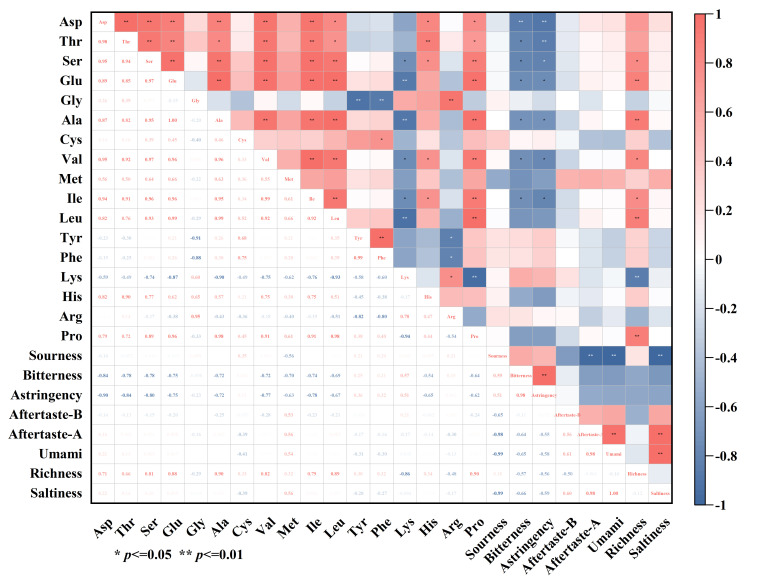
Spearman correlation analysis between amino acid composition and electronic tongue taste attributes. Red and blue colors indicate positive and negative correlations, respectively, and color intensity reflects the strength of the correlation coefficient. Asterisks indicate significant correlations (* *p* < 0.05, ** *p* < 0.01). Electronic tongue attributes include sourness, bitterness, astringency, aftertaste-B (bitterness aftertaste), aftertaste-A (astringency aftertaste), umami, richness, and saltiness.

Spearman correlation analysis was performed (*n* = 5). Bitterness and astringency showed significant negative correlations with Ile, Val, Ala, Glu, Ser, Thr, and Asp (*p* < 0.05; Figure 5). Asp exhibited the strongest inverse relationships (bitterness: r = −0.84; astringency: r = −0.90). These findings suggest that germination-induced changes in amino acid composition were associated with reduced bitterness- and astringency-related responses. Previous studies have shown that umami amino acids may suppress bitterness through taste–taste interactions and non-covalent interactions with bitter compounds [94,95]. In the present study, the strong negative correlations of Asp and Glu with bitterness-related responses support the view that these amino acids may contribute to taste modulation in germinated *Coix*. Interestingly, although several canonical bitter amino acids were present, only Val and Ile showed negative correlations with bitterness in the present dataset. This indicates that bitterness perception in *Coix* was not determined simply by the total amount of bitter amino acids, but rather by the overall composition and interaction of taste-active metabolites. Taken together, the amino acid results suggest that germination-induced taste improvement was not simply a consequence of reducing bitter compounds, but also of reshaping the amino acid matrix in a direction favorable for umami enhancement. In particular, Glu appears to serve as a central metabolic node, because it is directly involved in GABA biosynthesis while also contributing strongly to umami perception. Asp showed the strongest negative correlation with bitterness and astringency, whereas Glu appeared to be associated with both nutritional enhancement and flavor improvement. These results indicate that amino acid metabolism during germination is closely linked to both functional component accumulation and taste modulation in *Coix*.

### 3.13. Correlation Analysis Between Fatty Acids and Taste Properties

**Figure 6:**
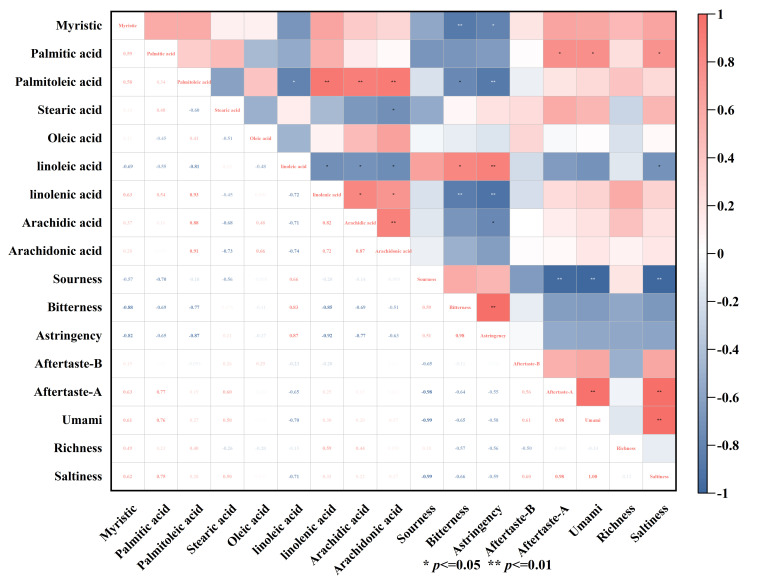
Spearman correlation analysis between fatty acid composition and electronic tongue taste attributes. Red and blue colors indicate positive and negative correlations, respectively, and color intensity reflects the strength of the correlation coefficient. Asterisks indicate significant correlations (* *p* < 0.05, ** *p* < 0.01). Electronic tongue attributes include sourness, bitterness, astringency, aftertaste-B (bitterness aftertaste), aftertaste-A (astringency aftertaste), umami, richness, and saltiness.

As shown in Figure 6, Linoleic acid was positively correlated with bitterness and astringency (r = 0.83 and 0.87, respectively; *p* < 0.05) and negatively correlated with saltiness (r = −0.71, *p* < 0.05). Bitterness and astringency were negatively correlated with Myristic, Palmitic, and Linolenic acids (*p* < 0.05). Among them, Myristic acid and Linolenic acid showed highly significant negative correlations with bitterness (r = −0.88 and −0.85, respectively; *p* < 0.01). Palmitic acid was positively correlated with saltiness, umami, and aftertaste A. Arachidic acid was negatively correlated with astringency (r = −0.77, *p* < 0.05). These results indicate that germination-related remodeling of fatty acid composition was associated with measurable changes in taste-related responses. Previous studies have suggested that debittering or ripening processes can alter fatty acid composition in parallel with bitterness changes in plant foods [96,97]. In the present study, linoleic acid decreased after germination, whereas myristic acid, palmitoleic acid, linolenic acid, and arachidic acid appeared or increased. When considered together with the amino acid correlation results, the fatty acid data indicate that bitterness reduction in germinated *Coix* was associated with coordinated changes in both nitrogenous and lipid-related taste contributors. Specifically, the decrease in linoleic acid, which showed a positive correlation with bitterness and astringency, occurred concurrently with increased levels of Asp and Glu, which were negatively associated with bitterness-related responses. This combined pattern suggests that taste improvement after germination was not driven by a single compound class, but by a broader metabolic reorganization involving both fatty acid remodeling and amino acid-mediated taste compensation.

## 4. Conclusions

This study systematically compared water immersion germination, ultrasound-assisted immersion germination, and heat-treated immersion germination in terms of their effects on the nutritional and sensory quality of *Coix*, and further analyzed the relationships among post-germination amino acids, fatty acids, and electronic tongue responses. The results showed that the three germination strategies exerted distinct regulatory effects. Ultrasound-assisted immersion was more favorable for GABA and amino acid accumulation, whereas heat-treated immersion was more effective in reducing phytic acid. Among the tested treatments, UI-20 performed best in increasing GABA and total amino acids while reducing bitterness, HI-40 showed relatively high protein, dietary fiber, and antioxidant activity, HI-50 exhibited the highest germination rate, and HI-60, although effective in reducing phytic acid, impaired seed vitality. Correlation analysis further suggested that the reduction in bitterness and astringency after germination may be associated with increased Asp and Glu together with decreased linoleic acid, indicating that germination can improve both the nutritional quality and sensory acceptability of *Coix* to some extent. The significance of this work lies in evaluating different germination strategies from the dual perspectives of nutritional enhancement and palatability improvement, thereby providing an experimental basis for the use of *Coix* as a functional grain ingredient.

These results indicate that the optimal pretreatment depends on the target quality attribute. Based on the above findings, germination methods and conditions can be selected according to different requirements to achieve the targeted utilization of *Coix* seeds. For example, to maximize GABA and umami amino acids (e.g., for functional beverages and nutritional supplements), UI-20 is the preferred option, as this treatment yields the highest levels of GABA and total amino acids while achieving the most pronounced reduction in bitterness. However, the germination rate under this treatment is moderate. Therefore, it is suitable for production processes where seed vigor is not the ultimate goal. To minimize phytic acid content (e.g., for infant cereals and mineral-fortified products), HI-60 can be selected. If the target product requires high germination activity (e.g., for live sprout production or seedling propagation), HI-60 is not recommended; in such cases, HI-50 is the preferred alternative. HI-40 can be chosen for high-dietary-fiber products. Overall, germination represents a feasible strategy for improving the nutritional value and sensory acceptability of *Coix*. The limitation of this study is the inability to identify specific bitter peptides and individual bitter polyphenols. At the same time, time zero control should be added in each preprocessing step to further distinguish between immediate preprocessing effects and subsequent germination effects. Future work should combine targeted bitterness-compound identification, enzyme assays, gene expression analyses (e.g., qPCR for GAD, phytase, and desaturase genes), and evaluation of seed coat permeability to clarify the molecular basis by which different physical pretreatments regulate the nutritional and sensory properties of *Coix*, and to support further process optimization and product application.

## Figures and Tables

**Table 1:** The effect of different germination conditions on the amino acid content of *Coix*.

Amino Acids	Amino Acid Content (mg/g)							
	NG	WI	UI-10	UI-15	UI-20	HI-40	HI-50	HI-60
Asp	6.96 ± 2.25 ^b^	9.10 ± 0.37 ^a^	9.95 ± 0.47 ^a^	9.89 ± 0.19 ^a^	10.02 ± 0.30 ^a^	9.31 ± 0.12 ^a^	9.43 ± 0.20 ^a^	9.46 ± 0.14 ^a^
Thr *	3.09 ± 0.71 ^b^	3.85 ± 0.18 ^a^	3.99 ± 0.20 ^a^	3.97 ± 0.07 ^a^	4.02 ± 0.16 ^a^	3.75 ± 0.03 ^a^	3.86 ± 0.06 ^a^	3.78 ± 0.07 ^a^
Ser	4.90 ± 1.17 ^c^	5.87 ± 0.15 ^b^	6.50 ± 0.31 ^ab^	6.66 ± 0.10 ^ab^	6.89 ± 0.10 ^a^	6.01 ± 0.09 ^ab^	6.19 ± 0.19 ^ab^	6.00 ± 0.06 ^b^
Glu	24.34 ± 6.92 ^d^	28.62 ± 0.66 ^cd^	36.03 ± 1.50 ^ab^	38.49 ± 0.76 ^a^	39.41 ± 2.02 ^a^	32.98 ± 0.48 ^bc^	32.84 ± 0.72 ^bc^	32.33 ± 0.37 ^bc^
Gly	3.67 ± 0.54 ^c^	4.82 ± 0.03 ^a^	4.03 ± 0.28 ^bc^	3.94 ± 0.20 ^bc^	3.92 ± 0.36 ^bc^	4.06 ± 0.01 ^b^	4.24 ± 0.01 ^b^	4.12 ± 0.07 ^bc^
Ala	10.38 ± 1.72 ^c^	11.57 ± 0.14 ^c^	14.43 ± 0.51 ^bc^	15.58 ± 0.41 ^b^	15.53 ± 0.50 ^b^	13.40 ± 0.13 ^c^	13.30 ± 0.24 ^c^	13.05 ± 0.11 ^c^
Cys	2.01 ± 0.11 ^abc^	1.92 ± 0.06 ^abc^	1.96 ± 0.12 ^abc^	2.13 ± 0.18 ^ab^	2.16 ± 0.09 ^a^	1.78 ± 0.07 ^bc^	1.88 ± 0.21 ^bc^	1.92 ± 0.04 ^abc^
Val *	5.47 ± 0.57 ^d^	6.44 ± 0.07 ^c^	7.07 ± 0.50 ^abc^	7.45 ± 0.41 ^a^	7.21 ± 0.37 ^ab^	6.74 ± 0.12 ^bc^	6.64 ± 0.04 ^bc^	6.66 ± 0.27 ^bc^
Met *	1.16 ± 0.85 ^b^	1.33 ± 0.47 ^b^	2.03 ± 0.47 ^ab^	1.42 ± 0.79 ^b^	2.71 ± 0.22 ^a^	1.79 ± 0.51 ^b^	1.12 ± 0.83 ^b^	1.78 ± 0.12 ^ab^
Ile *	3.30 ± 0.58 ^d^	4.16 ± 0.13 ^c^	4.67 ± 0.35 ^abc^	5.01 ± 0.17 ^a^	4.89 ± 0.17 ^ab^	4.47 ± 0.03 ^c^	4.22 ± 0.21 ^c^	4.39 ± 0.23 ^bc^
Leu *	13.49 ± 2.57 ^d^	14.42 ± 0.29 ^d^	19.31 ± 0.76 ^ab^	21.12 ± 0.44 ^a^	21.42 ± 1.11 ^a^	17.65 ± 0.17 ^bc^	17.25 ± 0.27 ^bc^	17.17 ± 0.12 ^c^
Tyr	6.55 ± 1.27 ^a^	4.86 ± 0.08 ^d^	6.01 ± 0.27 ^abc^	6.36 ± 0.12 ^ab^	6.21 ± 0.11 ^abc^	5.51 ± 0.13 ^cd^	5.39 ± 0.18 ^cd^	5.50 ± 0.06 ^bcd^
Phe *	8.03 ± 1.44 ^a^	6.17 ± 0.13 ^c^	7.39 ± 0.32 ^ab^	7.82 ± 0.20 ^ab^	7.88 ± 0.15 ^a^	6.80 ± 0.11 ^bc^	6.74 ± 0.18 ^bc^	6.78 ± 0.04 ^bc^
Lys *	3.67 ± 0.56 ^ab^	4.10 ± 0.08 ^a^	3.07 ± 0.10 ^cd^	2.75 ± 0.27 ^d^	2.73 ± 0.28 ^d^	3.13 ± 0.21 ^bc^	3.41 ± 0.05 ^bc^	3.37 ± 0.07 ^bc^
His	2.48 ± 0.36 ^b^	3.10 ± 0.09 ^a^	2.92 ± 0.28 ^a^	2.98 ± 0.09 ^a^	3.02 ± 0.16 ^a^	2.79 ± 0.09 ^ab^	2.85 ± 0.07 ^ab^	2.87 ± 0.08 ^a^
Arg *	6.10 ± 0.85 ^b^	7.90 ± 0.3 ^a^	6.34 ± 0.48 ^b^	6.17 ± 0.32 ^b^	5.99 ± 0.26 ^b^	6.23 ± 0.23 ^b^	6.69 ± 0.09 ^b^	6.45 ± 0.28 ^b^
Pro	7.88 ± 1.09 ^d^	8.16 ± 0.46 ^d^	11.58 ± 1.39 ^ac^	12.45 ± 0.30 ^a^	11.97 ± 1.77 ^ab^	10.61 ± 0.67 ^cd^	9.82 ± 0.16 ^cd^	9.77 ± 0.45 ^bcd^
Essential amino acids	40.71 ± 4.37 ^d^	43.60 ± 1.41 ^cd^	50.48 ± 2.68 ^ab^	52.55 ± 1.51 ^a^	53.78 ± 0.55 ^a^	47.16 ± 1.09 ^bc^	46.82 ± 0.43 ^bc^	46.11 ± 1.22 ^c^
Sum of amino acids	113.54 ± 17.29 ^d^	126.46 ± 3.05 ^cd^	147.35 ± 7.58 ^ab^	154.26 ± 3.60 ^a^	156.04 ± 4.29 ^a^	137.07 ± 2.48 ^bc^	135.44 ± 1.05 ^bc^	135.93 ± 2.26 ^bc^

All the results were expressed as mean values ± SD. Means with the different letters in the same row were significantly different (*p* < 0.05). NG: non-germinated *Coix*; WI: water immersion germinated *Coix*; UI-10, UI-15, UI-20: *Coix* germinated by ultrasound-assisted immersion for 10, 15, and 20 min, respectively; HI-40, HI-50, HI-60: *Coix* germinated by soaking *Coix* in 40, 50, and 60 °C water for 15 min, respectively. * Representing essential amino acids.

**Table 2:** Amino acid taste activity value (TAV) under different germination conditions.

Flavor Amino Acid	Taste Characteristics	Taste Threshold	TAV							
			NG	WI	UI-10	UI-15	UI-20	HI-40	HI-50	HI-60
Asp	Umami	1	6.96 ± 2.25 ^b^	9.10 ± 0.37 ^a^	9.95 ± 0.47 ^a^	9.89 ± 0.19 ^a^	10.02 ± 0.30 ^a^	9.31 ± 0.12 ^a^	9.43 ± 0.20 ^a^	9.46 ± 0.14 ^a^
Glu	Umami	0.3	81.13 ± 23.09 ^d^	95.40 ± 2.21 ^cd^	120.1 ± 5.01 ^b^	128.30 ± 2.53 ^a^	131.39 ± 6.75 ^a^	109.95 ± 1.61 ^abc^	109.48 ± 2.41 ^bc^	107.77 ± 1.23 ^bc^
Thr	Sweetness	2.6	1.19 ± 0.27 ^b^	1.48 ± 0.07 ^a^	1.53 ± 0.08 ^a^	1.52 ± 0.02 ^a^	1.55 ± 0.06 ^a^	1.44 ± 0.01 ^a^	1.48 ± 0.02 ^a^	1.43 ± 0.01 ^a^
Ser	Sweetness	1.5	3.27 ± 0.78 ^c^	3.91 ± 0.10 ^b^	4.33 ± 0.20 ^ab^	4.44 ± 0.07 ^ab^	4.59 ± 0.07 ^a^	4.00 ± 0.06 ^b^	4.13 ± 0.13 ^ab^	4.00 ± 0.04 ^b^
Pro	Sweetness	3	2.63 ± 0.36 ^d^	2.72 ± 0.15 ^cd^	3.86 ± 0.46 ^ab^	4.15 ± 0.10 ^a^	3.99 ± 0.59 ^a^	3.53 ± 0.22 ^ab^	3.27 ± 0.05 ^bc^	3.26 ± 0.15 ^bcd^
Gly	Sweetness	1.3	2.83 ± 0.42 ^c^	3.71 ± 0.02 ^a^	3.10 ± 0.22 ^bc^	3.03 ± 0.15 ^bc^	3.01 ± 0.28 ^bc^	3.12 ± 0.01 ^bc^	3.26 ± 0.01 ^b^	3.17 ± 0.06 ^bc^
Ala	Sweetness	0.6	17.24 ± 2.92 ^d^	19.29 ± 0.24 ^d^	24.05 ± 0.86 ^ab^	25.97 ± 0.69 ^a^	25.89 ± 0.83 ^a^	22.34 ± 0.22 ^bc^	22.17 ± 0.40 ^bc^	21.70 ± 0.13 ^c^
Val	Bitterness	0.4	13.69 ± 1.42 ^d^	16.11 ± 0.17 ^c^	17.69 ± 1.25 ^abc^	18.63 ± 1.04 ^a^	18.02 ± 0.94 ^ab^	16.86 ± 0.30 ^bc^	16.61 ± 0.11 ^bc^	16.67 ± 0.68 ^bc^
Met	Bitterness	0.3	3.90 ± 2.87 ^b^	4.43 ± 1.58 ^bc^	6.77 ± 1.57 ^abc^	4.75 ± 2.63 ^b^	9.05 ± 0.75 ^a^	5.99 ± 1.72 ^abc^	3.75 ± 2.77 ^bc^	5.95 ± 0.40 ^abc^
Ile	Bitterness	0.9	3.67 ± 0.65 ^d^	4.62 ± 0.14 ^c^	5.19 ± 0.38 ^abc^	5.57 ± 0.19 ^a^	5.43 ± 0.18 ^ab^	4.97 ± 0.04 ^abc^	4.69 ± 0.24 ^c^	4.88 ± 0.25 ^bc^
Leu	Bitterness	1.9	7.10 ± 1.35 ^d^	7.59 ± 0.15 ^d^	10.17 ± 0.39 ^ab^	11.12 ± 0.23 ^a^	11.27 ± 0.58 ^a^	9.29 ± 0.08 ^bc^	9.08 ± 0.14 ^bc^	9.04 ± 0.06 ^c^
Phe	Bitterness	0.9	8.93 ± 1.60 ^a^	6.86 ± 0.14 ^d^	8.22 ± 0.36 ^abc^	8.69 ± 0.22 ^abc^	8.76 ± 0.17 ^ab^	7.56 ± 0.12 ^bcd^	7.49 ± 0.20 ^cd^	7.54 ± 0.05 ^cd^
His	Bitterness	0.2	12.40 ± 1.81 ^b^	15.51 ± 0.45 ^a^	14.63 ± 1.40 ^a^	14.90 ± 0.49 ^a^	15.13 ± 0.83 ^a^	13.98 ± 0.46 ^ab^	14.26 ± 0.35 ^ab^	14.36 ± 0.4 ^a^
Arg	Bitterness	0.5	12.21 ± 1.71 ^b^	15.81 ± 0.60 ^a^	12.69 ± 0.96 ^b^	12.34 ± 0.64 ^b^	11.99 ± 0.53 ^b^	12.46 ± 0.47 ^b^	13.39 ± 0.18 ^b^	12.90 ± 0.57 ^b^

Different letters in the same subgraph indicated significant differences (*p* < 0.05).

**Table 3:** The effect of different germination conditions on the fatty acid content of *Coix*.

Fatty Acid	Composition and Content of Fatty Acid (%)							
	NG	WI	UI-10	UI-15	UI-20	HI-40	HI-50	HI-60
Myristic *	ND	0.022 ± 0.005 ^b^	0.036 ± 0.008 ^ab^	0.028 ± 0.005 ^ab^	0.113 ± 0.127 ^a^	0.060 ± 0.008 ^ab^	0.055 ± 0.013 ^ab^	0.056 ± 0.010 ^ab^
Palmitic acid *	14.902 ± 0.121 ^f^	14.977 ± 0.032 ^f^	15.478 ± 0.007 ^e^	14.727 ± 0.021 ^g^	15.828 ± 0.015 ^d^	16.410 ± 0.015 ^b^	16.778 ± 0.022 ^a^	16.286 ± 0.013 ^c^
Palmitoleic acid	ND	0.097 ± 0.011 ^a^	0.078 ± 0.012 ^a^	0.082 ± 0.009 ^a^	0.093 ± 0.017 ^a^	0.085 ± 0.009 ^a^	0.078 ± 0.006 ^a^	0.089 ± 0.009 ^a^
Stearic acid *	2.237 ± 0.135 ^a^	1.927 ± 0.008 ^e^	2.025 ± 0.006 ^cd^	1.936 ± 0.002 ^de^	2.095 ± 0.008 ^bc^	2.139 ± 0.006 ^b^	2.137 ± 0.011 ^b^	2.148 ± 0.008 ^ab^
Oleic acid	45.677 ± 0.127 ^f^	47.087 ± 0.012 ^a^	46.377 ± 0.016 ^e^	46.625 ± 0.018 ^d^	46.755 ± 0.019 ^c^	46.214 ± 0.035 ^f^	44.446 ± 0.026 ^g^	46.887 ± 0.029 ^b^
Linoleic acid	37.181 ± 0.221 ^a^	34.999 ± 0.035 ^d^	35.078 ± 0.022 ^d^	35.768 ± 0.008 ^b^	34.317 ± 0.043 ^e^	34.190 ± 0.025 ^e^	35.580 ± 0.103 ^c^	33.684 ± 0.015 ^f^
Linolenic acid	ND	0.500 ± 0.002 ^c^	0.509 ± 0.021 ^c^	0.498 ± 0.032 ^c^	0.559 ± 0.003 ^b^	0.561 ± 0.000 ^b^	0.622 ± 0.002 ^a^	0.496 ± 0.001 ^c^
Arachidic acid *	ND	0.259 ± 0.008 ^b^	0.327 ± 0.027 ^a^	0.259 ± 0.007 ^b^	0.231 ± 0.020 ^b^	0.253 ± 0.038 ^b^	0.181 ± 0.029 ^c^	0.247 ± 0.030 ^b^
Arachidonic acid	ND	0.124 ± 0.043 ^a^	0.086 ± 0.037 ^ab^	0.083 ± 0.019 ^ab^	0.079 ± 0.014 ^ab^	0.081 ± 0.011 ^ab^	0.054 ± 0.014 ^b^	0.099 ± 0.040 ^ab^

All the results were expressed as mean values ± SD. ND represents undetected. Means with the different letters in the same row were significantly different (*p* < 0.05). NG: non-germinated *Coix*; WI: water immersion germinated *Coix*; UI-10, UI-15, UI-20: *Coix* germinated by ultrasound-assisted immersion for 10, 15, and 20 min, respectively; HI-40, HI-50, HI-60: *Coix* germinated by soaking *Coix* in 40, 50, and 60 °C water for 15 min, respectively. * Representing saturated fatty acids.

**Table 4:** Electronic tongue results under different germination conditions.

	Sourness	Bitterness	Astringency	Umami	Richness	Saltiness
NG	−38.56 ± 0.48 ^a^	−4.11 ± 1.08 ^a^	−16.93 ± 0.68 ^a^	21.21 ± 0.42 ^ab^	−0.02 ± 0.02 ^b^	35.04 ± 0.47 ^a^
WI	−37.98 ± 0.89 ^a^	−5.85 ± 1.42 ^ab^	−21.14 ± 3.13 ^a^	21.28 ± 0.66 ^ab^	−0.02 ± 0.03 ^b^	35.03 ± 1.00 ^a^
UI-10	−40.04 ± 2.69 ^a^	−7.33 ± 2.15 ^ab^	−22.86 ± 4.64 ^a^	22.50 ± 1.85 ^ab^	0.11 ± 0.09 ^ab^	37.15 ± 2.95 ^a^
UI-15	−35.83 ± 4.79 ^a^	−6.24 ± 2.63 ^ab^	−21.65 ± 4.97 ^a^	19.55 ± 3.12 ^b^	0.19 ± 0.35 ^a^	32.56 ± 5.19 ^a^
UI-20	−40.68 ± 2.26 ^a^	−8.34 ± 2.12 ^b^	−24.34 ± 5.05 ^a^	22.98 ± 1.59 ^ab^	0.14 ± 0.14 ^ab^	37.93 ± 2.64 ^a^
HI-40	−40.35 ± 2.64 ^a^	−7.47 ± 1.49 ^ab^	−23.36 ± 4.06 ^a^	22.89 ± 1.76 ^ab^	0.10 ± 0.09 ^ab^	37.76 ± 2.93 ^a^
HI-50	−39.16 ± 1.93 ^a^	−7.35 ± 1.52 ^ab^	−23.08 ± 3.78 ^a^	22.20 ± 1.32 ^ab^	0.14 ± 0.08 ^ab^	36.51 ± 2.15 ^a^
HI-60	−41.84 ± 2.82 ^a^	−7.59 ± 1.53 ^ab^	−23.71 ± 3.63 ^a^	23.74 ± 1.73 ^a^	0.03 ± 0.12 ^ab^	39.09 ± 2.99 ^a^

Results were expressed as mean ± standard deviation and lowercase letters within the same column were significantly different (*p* < 0.05). NG: non-germinated *Coix*; WI: water immersion germinated *Coix*; UI-10, UI-15, UI-20: *Coix* germinated by ultrasound-assisted immersion for 10, 15, and 20 min, respectively; HI-40, HI-50, HI-60: *Coix* germinated by soaking *Coix* in 40, 50, and 60 °C for 15 min.

## Data Availability

The original contributions presented in the study are included in the article; further inquiries can be directed to the corresponding authors.
